# Supplementary material for: Ultrasound pupillometry for the detection of a relative afferent pupillary defect (RAPD): Systematic evaluation in patients with optic neuritis and comparison with infrared video pupillometry
Source: PLoS One. 2025 Jan 10;20(1):e0315712. doi: 10.1371/journal.pone.0315712 (PMC11723539; doi:10.1371/journal.pone.0315712)
Supplement: Attachment — Submitted filename: 240331_PLOS_Review.docx [file pone.0315712.s002.docx]

PLOS ONE – PONE-D-24-06833

It has been a pleasure to review the authors’ manuscript. In this exploratory experiment, the authors evaluated the optic neuritis (ON) diagnostic utility of B-mode ultrasound pupillometry (UP) against infrared video pupillometry (IVP) across multiple visual endpoints of ON. The authors concluded that UP performed similarly to IVP and RAPD assessment by UP may detect subclinical ON damage in patients with multiple sclerosis. They also offered a standardized protocol for RAPD detection by UP in routine clinical evaluation. Multiple merits are present in the manuscript including an interesting experimental design, a coherent introduction section, detailed descriptions of the methods and report of the results, as well as a thorough discussion of the limitations. However, several areas of improvement need to be addressed until I endorse the publication.

Major points:

- The first paragraph in the introduction section has a strong flow. To aid in readers’ understanding, could the authors include a brief review of how the gold standard IVP has been applied to detect ON? Additional background information on the technical differences and general pros and cons in the clinical settings between IVP and UP in measuring PLR would be valuable (even though some information was discussed in the discussion section). Please also highlight the benefits of discussing other visual endpoints of ON (including both objective and subjective measures).
- Line 475: Could the authors elaborate on the possible factors in the higher detection sensitivity of UP?
- In addition to the quality plots, as part of good practice in pupillometry studies, could the authors also provide the aggregated waveform of pupillary changes during the tests?

Minor points:

- Line 81-82: there appears to be only one sentence in a paragraph.
- Line 85 (citation 12): please provide the correlation value of the strong correlation as a reference for the effect size in the present study.
- Line 115: did the authors also control for the use of stimulants as caffeine in the sample?
- Line 155: please provide one sample item from the questionnaire and report the likert scale as well as its psychometric properties based on literature. Please also state internal reliability as Cronbach’s alpha.
- Line 190-191: please discuss the exploratory nature of this study in the introduction.
- Could the authors also provide reference values of the key ON related measures (e.g., PCT) from much larger datasets for readers to compare with the sample in this study?
