# Supplementary material for: Ultrasound pupillometry for the detection of a relative afferent pupillary defect (RAPD): Systematic evaluation in patients with optic neuritis and comparison with infrared video pupillometry
Source: PLoS One. 2025 Jan 10;20(1):e0315712. doi: 10.1371/journal.pone.0315712 (PMC11723539; doi:10.1371/journal.pone.0315712)
Supplement: Attachment — Submitted filename: correction.docx [file pone.0315712.s003.docx]

20^th^ April, 2024
PLOS ONE Journal

Dear Editor-in-chief,
I am enclosing herewith ‘reviewer attachments’ for Manuscript Number PONE-D-24-06833 and manuscript entitled “Ultrasound pupillometry for the detection of a relative afferent pupillary defect (RAPD): systematic evaluation in patients with optic neuritis and comparison with infrared video pupillometry” for peer review. The study presented favorable findings and could be considered acceptable with slight revisions as following:

1. The subheading "Swinging flashlight test" and "Ultrasound pupillometry vs. infrared video pupillometry" in the results section (line 226 and 231 respectively) lack clarity regarding the type of analysis conducted.
2. Table 2 fails to include the p-value for the correlation coefficient between UP and IVP.
3. The legend of Figure 4 does not specify the pupillometry method used to measure the pupillary diameter of ON- eyes.
4. The legend of Figure 5 does not provide an explanation for the basis of the study groups.
5. Line 323-327 presents a detailed analysis of five MS ON- patients with CR>1.3. However, in lines 313-314, it mentions ten MS ON- patients with CR>1.2, requiring clarification.
6. Line 343, regarding "Pupil constriction time," lacks an explanation of the analysis under the subheading.
7. There are multiple figures which can be combines together as subsections of a figure legend.
8. A subheading for the conclusion is missing.
9. The methodology section lacks clarity regarding the subheading "Clinical examination protocol" following the discussion section of the manuscript.
10. There are a few typos, grammar, and phrasing issues.

I hope these suggestions will assist with the peer review of this manuscript. Please don't hesitate to reach out if you require any further assistance.

Thanks and regards

Dr Priyanka

M.D. (Emergency Medicine)

AIIMS, Delhi, India
