# Supplementary material for: Ultrasound pupillometry for the detection of a relative afferent pupillary defect (RAPD): Systematic evaluation in patients with optic neuritis and comparison with infrared video pupillometry
Source: PLoS One. 2025 Jan 10;20(1):e0315712. doi: 10.1371/journal.pone.0315712 (PMC11723539; doi:10.1371/journal.pone.0315712)
Supplement: Attachment — Submitted filename: Response to Reviewers.docx [file pone.0315712.s004.docx]

"Ultrasound pupillometry for the detection of a relative afferent pupillary defect (RAPD): systematic evaluation in patients with optic neuritis and comparison with infrared video pupillometry"

Manuscript ID: PONE-D-24-06833

**Response to Reviewers**

**Reviewer #1:**

**Reviewer’s point:**

It has been a pleasure to review the authors’ manuscript. In this exploratory experiment, the authors evaluated the optic neuritis (ON) diagnostic utility of B-mode ultrasound pupillometry (UP) against infrared video pupillometry (IVP) across multiple visual endpoints of ON. The authors concluded that UP performed similarly to IVP and RAPD assessment by UP may detect subclinical ON damage in patients with multiple sclerosis. They also offered a standardized protocol for RAPD detection by UP in routine clinical evaluation. Multiple merits are present in the manuscript including an interesting experimental design, a coherent introduction section, detailed descriptions of the methods and report of the results, as well as a thorough discussion of the limitations.

**Response**:

We would like to thank the Reviewer for this comment.

**Reviewer’s point:**

The first paragraph in the introduction section has a strong flow. To aid in readers’ understanding, could the authors include a brief review of how the gold standard IVP has been applied to detect ON?

**Response**:

We appreciate this thoughtful feedback. In response to this point, we added the following sentences as well as two additional references, i.e. Blazcek et al. (2012) and Cohen et al. (2015), to the introduction section:

"Infrared video pupillometry (IVP), the current gold standard for objective assessment of the PLR, provides precise quantitative measurements and allows for the analysis of the PLR through high-resolution video imaging [11]. For IVP, the infrared video camera records the PLR of the ipsilateral open eye after an Lstim of the integrated light diode with a fixed distance between the eye and the light source. IVP has previously been applied for the detection of an RAPD in patients with ON where it was shown to detect an RAPD with high sensitivity and specificity [12, 13]." (pages 5-6, lines 86-95)

**Reviewer’s point:**

Additional background information on the technical differences and general pros and cons in the clinical settings between IVP and UP in measuring PLR would be valuable (even though some information was discussed in the discussion section).

**Response**:

We appreciate this suggestion. To provide more background information on the technical differences and general pros and cons in the clinical settings between IVP and UP in measuring PLR we have added the following sentence to the introduction:

"In UP, patients are examined with their eyes closed to prevent contact of the ultrasound gel with the eye, while the examiner uses an external manual light source (penlight)." (page 5, lines 82-84)

Furthermore, we added the following passage:

"However, unlike ultrasound, IVP devices are rarely available in non-specialized clinical settings. Furthermore, IVP assessment requires the ability to fixate on a target and to keep the eyes open throughout the examination, whereas UP does not require patient cooperation." (page 6, lines 95-98)

**Reviewer’s point:**

Please also highlight the benefits of discussing other visual endpoints of ON (including both objective and subjective measures).

**Response:**

We thank the Reviewer for this comment. In response, we added the following passage to the discussion section:

"Among the strengths of this study is the inclusion of various objective (VA, OCT) and subjective (VQoL) visual outcome parameters for ON. VQoL is a subjective measure that captures the patient's perspective on the impact of visual impairments on their daily life and overall well-being. The correlation of UP and IVP parameters with VQoL therefore indicates that these parameters may reflect the real-world impact of ON on a patient’s daily life. Changes in the pRNFLT, as measured by OCT, indicates structural optic nerve damage. Altogether, our multifaceted approach enabled cross-validation of UP and IVP findings with other established objective and subjective outcome measures of ON, enhancing the clinical relevance of our findings." (page 27, lines 568-576)

**Reviewer’s point:**

Line 475: Could the authors elaborate on the possible factors in the higher detection sensitivity of UP?

**Response:**

Yes, we have now included the following sentences in the discussion section:

"UP is an objective and precise measuring tool, providing consistent and reliable measurements, whereas the SFLT relies on subjective visual evaluation by the examiner. In the SFLT, results depend on the examiner’s experience and judgment. In contrast, UP can detect subtle changes in pupil diameter that might be overlooked during the SFLT. Also, UP offers the possibility to document and save results on frozen images and video sequences of the PLR, which allows for re-analysis. All these factors may contribute to the higher detection sensitivity of UP for an RAPD." (page 25, lines 532-538)

**Reviewer’s point:**

In addition to the quality plots, as part of good practice in pupillometry studies, could the authors also provide the aggregated waveform of pupillary changes during the tests?

**Response**:

We concur with the Reviewer's assertion that the aggregation of pupillary changes during the tests would be interesting. However, in this study, we employed pupillometry assessments by ultrasound pupillometry (UP) and infrared video pupillometry (IVP) to measure two metric values: the maximum pupil diameter (PD) at rest and the minimum PD after a light stimulus. As a result, we did not record continuous data, which precluded analyses of the aggregated waveform of pupillary changes.

**Reviewer’s point:**

Line 81-82: there appears to be only one sentence in a paragraph.

**Response**:

We thank the Reviewer for bringing this formatting issue to our attention. To provide further clarification regarding the technical application of ultrasound pupillometry (UP), we have added another sentence to the respective paragraph.

“In UP, patients are examined with their eyes closed to prevent contact of the ultrasound gel with the eye, while the examiner uses an external manual light source (penlight).” (page 5, lines 82-84)

**Reviewer’s point:**

Line 85 (citation 12): please provide the correlation value of the strong correlation as a reference for the effect size in the present study.

**Response:**

We agree with the Reviewer that the actual correlation coefficient is a crucial information. We now included the correlation coefficient from the mentioned study (Farina et al., 2021):

“(Pearson’s correlation coefficient, *r*=0.831, *p* < 0.01)” Page 6, lines 98-99

**Reviewer’s point:**

Line 115: did the authors also control for the use of stimulants as caffeine in the sample?

**Response**:

We thank the Reviewer for this very helpful comment. In this study, we could not systematically control for potential effects of stimulants, such as caffeine or nicotine, on the pupillary reaction. However, while this is a possible limitation of our study, we consider it unlikely that any possible use of stimulants might have significantly affected the results of our study, as the RAPD detects relative differences of the pupillary reaction of both eyes. While stimulants could possibly affect pupillary reactions globally, they appear unlikely to affect relative differences of pupillary reactions of both eyes.

In response to this point, we added the following to the limitation section of the discussion:

„Another possible limitation of our study is that we could not systematically control for potential effects of stimulants, such as caffeine or nicotine, on the pupillary reaction. However, we consider it unlikely that any possible use of stimulants might have significantly affected the results of our study, as the RAPD detects relative differences of the pupillary reaction of both eyes. While stimulants could possibly affect pupillary reactions globally, they appear unlikely to affect relative differences of pupillary reactions of both eyes.” (page 27, line 580-586)

**Reviewer’s point:**

Line 155: please provide one sample item from the questionnaire and report the Likert scale as well as its psychometric properties based on literature.

**Response**:

According to the Reviewer’s request, we now provide a sample question from the NEI-VFQ and report the Likert scale as well as its psychometric properties based on literature (Mangione et al., 1998) in the revised manuscript:

“The NEI-VFQ is a reliable and valid tool to assess vQoL in a variety of chronic eye conditions. Its strong psychometric properties make it suitable for group-level comparisons in clinical research, offering precise and consistent evaluation of how visual impairment affects the patients’ daily life [22]. To provide an understanding of the items included in the NEI-VFQ, we here present a sample question and the Likert scale of the possible responses. Question: How much difficulty do you have reading street signs or the names of stores? Answers: No difficulty at all, a little difficulty, moderate difficulty, extreme difficulty, stopped doing this because of your eyesight, stopped doing this for other reasons or not interested in doing this." (page 9, lines 179-187)

Furthermore, we specified that the questionnaire utilized in this study was the German adaptation (Franke et al. 1998) of the National Eye Institute-Visual Function Questionnaire:

“We assessed vQoL using the German adaptation [19] of the National Eye Institute-Visual Function Questionnaire (NEI-VFQ) [20].” (page 9, lines 177-178)

**Reviewer’s point:**

Please also state internal reliability as Cronbach’s alpha.

**Response**:

We thank the Reviewer for suggesting to use this statistical measure to state internal reliability of the NEI-VFQ. In response, we added the following sentence to the statistics section:

“For internal reliability analysis of the vQoL, we calculated Cronbach’s alpha to assess the internal reliability of the NEI-FVQ.” (page 10, lines 219-221)

Additionally, in the results section, we stated the internal reliability as Cronbach’s alpha by including the following sentence:

“The results of the NEI-VFQ, assessing the vQoL, showed very strong internal reliability (Cronbach’s alpha=0.968).” (page 22, lines 436-437)

**Reviewer’s point:**

Line 190-191: please discuss the exploratory nature of this study in the introduction.

**Response**:

We agree with the Reviewer's recommendation to mention the exploratory nature of this cross-sectional observational study. To address this point, we have added the following statement to the introduction:

“In this exploratory study, we systematically evaluated UP for the detection of an RAPD in patients with ON, including a comparison with IVP.” (page 6, lines 103-104)

**Reviewer’s point:**

Could the authors also provide reference values of the key ON related measures (e.g., PCT) from much larger datasets for readers to compare with the sample in this study?

**Response**:

To address this point, we performed a literature search, which, however, did not identify large and comprehensive datasets that could provide reference values for the PCT. The largest currently available set of reference values for UP is derived from our own previous study, which included a total study population of n=100. We now refer to these reference values in the following sentence:

“Also, PCT measurements of HC were in line with the reference values (mean, (SD) left eye: 970 (261.6) ms; right eye: 967 (220) ms) for healthy subjects from our previous study [9].” (page 25, lines 520-522)

**Reviewer #2 Response to ‘correction’ document:**

**Reviewer’s point:**

The subheading “Swinging flashlight test” and “Ultrasound pupillometry vs. infrared video pupillometry” in the results section (line 226 and 231 respectively) lack clarity regarding the type of analysis conducted.

**Response**:

We thank the Reviewer for identifying potential sources of ambiguity. In response to this point, we clarified the respective subheadings:

“Prevalence of an RAPD as assessed by the swinging flashlight test” (page 13, lines 264-265)

and

“Comparative analysis of pupil diameter as assessed by ultrasound pupillometry and infrared video pupillometry” (page 14, lines 270-271)

**Reviewer’s point:**

Table 2 fails to include the p-value for the correlation coefficient between UP and IVP.

**Response**:

We thank the Reviewer for bringing this to our attention. In response, we included the missing *p*-values in Table 2 (page 15).

**Reviewer’s point:**

The legend of Figure 4 does not specify the pupillometry method used to measure the pupillary diameter of ON- eyes.

**Response**:

We thank the Reviewer for raising this point. Of note, Bland-Altman plots are used to graphically illustrate the comparison of two different methods for measuring the same parameter. In this case, the pupil diameter (PD) is being measured by ultrasound pupillometry (UP) and by infrared video pupillometry (IVP). The x-axis reports the difference in PD measurements between the two methods (PD in IVP - PD in UP), while the y-axis shows the mean PD of both methods ((PD in IVP + PD in UP)/2).

To clarify this aspect, we have added both pupillometry assessments in the figure’s caption:

“Fig 4. Bland-Altman plot of pupil diameter as measured by UP and IVP after direct light stimulus in ON- eyes.” (page 17, lines 341-342)

**Reviewer’s point:**

The legend of Figure 5 does not provide an explanation for the basis of the study groups.

**Response**: As stated in the study's methodology section, the study population was divided into four groups: (1) patients with acute ON, (2) patients with past ON, (3) patients with MS without ON, and (4) healthy controls (HC). This division of the study population was applied consistently throughout all analyses, tables, and figures. To acknowledge that an RAPD is a sign of a unilateral ON, the second group (patients with past ON) was subdivided into two subgroups, as already stated on page 18, lines 363-366. In response to this comment, we have included an explanatory sentence in the legend of figure 5:

“The x-axis represents the study groups: patients with acute ON (n=9), patients with past unilateral ON or bilateral ON with n(ON_L)≠n(ON_R) (n=26), patients with past bilateral ON with n(ON_L)=n(ON_R) (n=5), patients with MS without ON (n=31), and healthy controls (n=50).” (page 17, lines 361-364)

For consistency, we also included the following sentence to the figure’s legend:

“The y-axis represents the participant’s constriction ratio in UP.” (page 17, lines 364-365)

**Reviewer’s point:**

Line 323-327 presents a detailed analysis of five MS ON- patients with CR>1.3. However, in lines 313-314, it mentions ten MS ON- patients with CR>1.2, requiring clarification.

**Response**:

We agree that this statement might be confusing. We originally wanted to underline the fact that in our present study, all HCs CR were below CR=1.2, which shows that the CR=1.3 is a rather stringent cut-off value. In response to this comment, we deleted the potentially confusing sentence on page 17, line 356.

**Reviewer’s point:**

Line 343, regarding "Pupil constriction time," lacks an explanation of the analysis under the subheading.

**Response**:

We thank the Reviewer for drawing our attention to this possible source of inaccuracy and amended the subheading of the results section as follows:

“Comparison of pupil constriction time in eyes with ON and eyes of HC” (page 19, lines 393-394)

**Reviewer’s point:**

A subheading for the conclusion is missing.

**Response**: We are grateful for this comment. In response, we added a respective subheading to the discussion section:

“Conclusions” (page 28, line 595)

**Reviewer’s point:**

The methodology section lacks clarity regarding the subheading "Clinical examination protocol" following the discussion section of the manuscript.

**Response**:

We thank the Reviewer for raising this point. For clarification, we added the following sentence in the Methods section:

“To facilitate the introduction of UP into routine clinical practice, we developed a short clinical examination protocol, which we added as a separate section after the discussion.” (page 8, lines 152-154)

Also, we specified in the subheading that this clinical protocol refers to the use of UP.

“Clinical examination protocol for UP” (page 28, lines 605)

**Reviewer’s point:**

There are a few typos, grammar, and phrasing issues.

**Response**:

In response to the Reviewer’s comment the manuscript was edited by a native speaker. We trust that the revised version will meet the requisite standards of language and grammar.
