# Supplementary material for: Ultrasound pupillometry for the detection of a relative afferent pupillary defect (RAPD): Systematic evaluation in patients with optic neuritis and comparison with infrared video pupillometry
Source: PLoS One. 2025 Jan 10;20(1):e0315712. doi: 10.1371/journal.pone.0315712 (PMC11723539; doi:10.1371/journal.pone.0315712)
Supplement: Attachment — Submitted filename: plos review final.docx [file pone.0315712.s005.docx]

25^th^ October, 2024
PLOS ONE Journal

Dear Editor-in-chief,

I am enclosing herewith ‘reviewer attachments’ for Manuscript Number **PONE-D-24-06833R1** and manuscript entitled **“Ultrasound pupillometry for the detection of a relative afferent pupillary defect (RAPD): systematic evaluation in patients with optic neuritis and comparison with infrared video pupillometry”** for peer review. The study presented favorable findings and could be considered acceptable with slight revisions as following:

1. In the comment regarding page 6, lines 95-98: "The author states that 'UP does not require patient cooperation.' However, there is no discussion about the feasibility of UP in agitated patients. This statement is only partially correct, as UP does not fully eliminate the need for patient cooperation."
2. In the comment regarding page 25, lines 532-538: "The author has not provided citations to support the literature review in this section."

I hope these suggestions will assist with the peer review of this manuscript. Please don't hesitate to reach out if you require any further assistance.

Thanks and regards

Dr Priyanka

M.D. (Emergency Medicine)

SGT Medical College, Hospital and Research Institute, Gurugram, Haryana, India
