# Supplementary material for: Ultrasound pupillometry for the detection of a relative afferent pupillary defect (RAPD): Systematic evaluation in patients with optic neuritis and comparison with infrared video pupillometry
Source: PLoS One. 2025 Jan 10;20(1):e0315712. doi: 10.1371/journal.pone.0315712 (PMC11723539; doi:10.1371/journal.pone.0315712)
Supplement: Attachment — Submitted filename: Response to Reviewers.docx [file pone.0315712.s006.docx]

"Ultrasound pupillometry for the detection of a relative afferent pupillary defect (RAPD): systematic evaluation in patients with optic neuritis and comparison with infrared video pupillometry"

Manuscript ID: PONE-D-24-06833R2

**Response to Reviewers**

**Reviewer #1:**

**Reviewer’s point:**

The authors have thoroughly responded to and tried to address all my comments. Thank you for your effort!

**Response**:

We appreciate this comment.

**Reviewer #2:**

**Reviewer’s point:**

Dual Publication: Ensure that the findings and data presented in this manuscript have not been published elsewhere or submitted to another journal simultaneously, as this would constitute dual publication, which violates ethical standards in research publishing.

**Response**:

We herewith confirm that the findings and data presented in this manuscript are original and have not been published elsewhere or submitted to another journal simultaneously, as already stated in the original Cover Letter from February 2024. We are fully aware of and adhered to the ethical standards in research publishing, including the guidelines concerning dual publication, and we are committed to maintaining the integrity of the scientific literature.

**Reviewer’s point:**

Research Ethics: The author should confirm that all patient data was handled in accordance with ethical guidelines, including obtaining informed consent and ensuring patient confidentiality.

**Response**:

We herewith confirm that all patient data were handled in strict accordance with the ethical guidelines that govern our research. As already stated in the manuscript, written informed consent was obtained from all participants prior to inclusion into the study and patient confidentiality was ensured throughout the study. In response to the Reviewer’s comment, we have amended the respective passage in the Methods Section of the manuscript to mention more explicitly that patient confidentiality was ensured throughout the study:

“This cross-sectional observational study was approved by the Institutional Review Board of Charité – Universitätsmedizin Berlin (EA1/190/15) and was conducted in accordance with the current applicable ethical guidelines of the Declaration of Helsinki and German law. Written informed consent was obtained from all participants prior to inclusion into the study and patient confidentiality was ensured throughout the study.” (page 6, lines 110-114)

**Reviewer’s point:**

Publication Ethics: Proper credit should be given through citations to all relevant sources in the literature review. Furthermore, the manuscript should be checked for any potential conflicts of interest or ethical concerns.

**Response**:

We concur with the Reviewer's comment and thank them for raising this important point. We confirm that all relevant sources have been subjected to a comprehensive review and cited in accordance with the standards set forth in the literature review. In instances where citations were absent, we have duly added them. Furthermore, the manuscript has been meticulously examined for any potential conflicts of interest or ethical concerns, and all necessary disclosures have been included to ensure compliance with the standards set forth in the publication ethics code.

**Reviewer’s point:**

In the comment regarding page 6, lines 95-98: "The author states that 'UP does not require patient cooperation.' However, there is no discussion about the feasibility of UP in agitated patients. This statement is only partially correct, as UP does not fully eliminate the need for patient cooperation."

**Response**:

We thank the Reviewer for bringing this to our attention. In response, we included the following additional statement:

“(…) UP does not require active patient cooperation, provided that the patient is able to remain seated or lie quietly during the procedure.” (page 6, lines 95-96)

**Reviewer’s point:**

In the comment regarding page 25, lines 532-538: "The author has not provided citations to support the literature review in this section."

**Response**:

We thank the Reviewer for highlighting this important point. In response, we have incorporated the missing references into the manuscript (Schmidt et al. 2017; Broadway, 2012; Thompson et al. 1981; Beisse et al. 2020).

“UP is an objective and precise measuring tool, providing consistent and reliable measurements [9], whereas the SFLT relies on subjective visual evaluation by the examiner [6, 7]. In the SFLT, results depend on the examiner’s experience and judgment [8].” (page 25, lines 513-516)
